# Folate Attenuates Ulcerative Colitis via PI3K/AKT/NF-κB/MLCK Axis Inhibition to Restore Intestinal Barrier Integrity

**DOI:** 10.3390/biology14111573

**Published:** 2025-11-10

**Authors:** Shize Zhang, Tian Cheng, Yuang Chen, Mengqin Wang, Guangji Wang, Jiye Aa

**Affiliations:** Jiangsu Provincial Key Laboratory of Drug Metabolism and Pharmacokinetics, State Key Laboratory of Natural Medicines, China Pharmaceutical University, Nanjing 210009, China; marshzsz@126.com (S.Z.); 18739192744@163.com (T.C.); cyamail@163.com (Y.C.); 18362073305@163.com (M.W.)

**Keywords:** mendelian randomization, folate, ulcerative colitis, intestinal barrier, PI3K/AKT/NF-κB/MLCK/MLC2 axis

## Abstract

Vitamin homeostasis plays a critical role in inflammatory bowel disease management, yet the protective mechanisms and clinical utility of specific vitamins remain incompletely characterized. Within this context, a two-sample Mendelian randomization analysis leveraging genetic instruments for measuring circulating vitamin levels identified folate as a protective factor against ulcerative colitis (UC). To validate these findings, a DSS-induced colitis model was established with serial serum folate measurements. Therapeutic folate supplements were subsequently administered, followed by a comprehensive evaluation of epithelial barrier modulation through in vivo and recombinant TNF-α/IFN-γ-induced in vitro models. This included assessment of junctional proteins, ultrastructural analysis by transmission electron microscopy, and functional quantification of barrier integrity using transepithelial electrical resistance with paracellular permeability assays in epithelial monolayers. Molecular mechanisms were investigated through RNA sequencing complemented by immunoblot validation of key pathway components. The results demonstrated decreased serum folate levels in DSS-induced colitis mice, whereas folate supplementation ameliorated disease severity and attenuated intestinal inflammation and histopathological damage. Crucially, folate restored epithelial barrier structural integrity and function both in vivo and in vitro. Mechanistically, folate mediated barrier restoration through suppression of the PI3K/AKT/NF-κB/MLCK/MLC2 signaling axis. Collectively, the results of this study provide mechanistic insights that support the use of folate as an active therapeutic molecule in patients with UC.

## Simple Summary

Based on emerging evidence linking vitamin homeostasis to inflammatory bowel disease, this study investigated the causal role and therapeutic potential of folate in ulcerative colitis. By employing Mendelian randomization and experimental models, we demonstrated that folate supplementation markedly attenuated colitis severity, suppressed inflammatory responses, and restored the structure and function of the epithelial barrier. Mechanistically, folate acts through inhibition of the PI3K/AKT/NF-κB/MLCK/MLC2 signaling axis, leading to improved barrier integrity and reduced mucosal inflammation. These findings establish folate not only as a protective nutrient but also as a potential therapeutic agent, supporting its use in clinical nutritional strategies for ulcerative colitis management.

## 1. Introduction

Inflammatory bowel disease (IBD) comprises chronic, idiopathic, and progressive gastrointestinal disorders characterized by relapsing mucosal inflammation, primarily Crohn’s disease (CD) and ulcerative colitis (UC) [1,2]. Once considered a western-centric condition, the incidence of IBD is now rapidly increasing across Asia and other historically low-prevalence regions, evolving into a global health challenge [3,4,5,6]. However, the precise pathogenesis of IBD remains incompletely defined; the current consensus positions dysregulated mucosal immunity, barrier disruption, genetic susceptibility, and diet triggers as convergent drivers of disease [7,8]. This pathogenic complexity inevitably creates therapeutic dilemmas in clinical practice. Current interventions, including conventional small-molecule drugs, biologics, and novel targeted agents, achieve sustained clinical responses in only a fraction of patients [9,10]. Consequently, developing safer and more efficacious therapeutics is imperative to address critical gaps in current IBD management strategies.

Vitamins, as essential organic micronutrients acquired through dietary sources, function as noncaloric mediators of fundamental biological processes [11]. They play multiple roles, including acting as enzymatic cofactors in energy metabolism and nucleic acid biosynthesis [12,13], functioning as antioxidants that neutralize reactive oxygen species (ROS) to maintain cellular integrity [14], and regulating gene expression and epigenetic regulation by nuclear receptor-mediated pathways [15]. The globalization of IBD parallels dietary and lifestyle westernization, and emerging clinical research has revealed intricate bidirectional links between metabolic disorders and IBD pathogenesis [16,17]. This evidence collectively suggests that nutritional management represents a significant and modifiable element in IBD development and therapeutic approaches. Notably, several studies have revealed that specific vitamins play crucial roles in modulating disease progression. Among these, vitamin D is well established; in preclinical models, it exerts therapeutic effects by regulating mucosal immunity and barrier function through vitamin D receptors (VDRs) [18,19,20]. Additionally, deficiencies in vitamins B5 and B1 have been recognized as significant contributors to disease progression, but the therapeutic efficacy of exogenous supplementation remains insufficiently validated [21,22]. Despite these observations, the absence of mechanistic validation and robust clinical evidence precludes definitive causal links between specific vitamin deficiencies and IBD, limiting clinical translation.

Mendelian randomization (MR) is an analytical approach that uses genetic variants as instrumental variables to infer causal relationships between modifiable exposures and disease risk [23]. MR relies on three core assumptions: genetic instruments must be strongly associated with target exposures, maintain independence from confounding factors, and influence outcomes exclusively through the specified exposure pathway [24,25]. This framework effectively minimizes confounding bias and reverse causation. Building on this foundation, this study applied MR to infer causal effects of circulating vitamin profiles on IBD risk, followed by experimental validation in preclinical models and mechanistic analyses, thereby providing novel etiological insights and actionable strategies for patient management.

## 2. Materials and Methods

### 2.1. Two-Sample Mendelian Randomization Analysis

Genetic instruments for circulating vitamins were obtained from published genome-wide association studies (GWAS): Vitamin A [26], B9 and B12 [27], D [28], E [29], B6 [30], and C [31]. Details of SNPs were listed in Supplementary Table S1. Outcome data for inflammatory bowel disease were obtained from the UK Biobank using International Classification of Diseases, Tenth Revision (ICD-10) codes K50-K51 in combination with self-reported diagnoses. The analysis included 1041 cases of Crohn’s disease with 461,532 controls and 2569 cases of ulcerative colitis with 453,779 controls.

Two-sample MR was performed using inverse-variance weighted (IVW) regression as primary analysis, with sensitivity analyses via MR-Egger and weighted median methods. The results of the sensitivity analyses are provided in Supplementary Figures S1–S6. All SNPs met genome-wide significance (*p* < 5 × 10^−8^), were clumped (r^2^ < 0.001), and passed F-statistic > 10 validation to ensure robust causal inference. All analyses were conducted in R v4.3.1 using the TwoSampleMR package (v0.5.7).

### 2.2. Animals

8-week-old male C57BL/6J mice were purchased from Vital River Laboratories (Beijing, China). Mice were maintained under specific pathogen-free (SPF) conditions with a 12 h light/dark cycle in a thermally regulated environment, provided ad libitum access to food and water. All experimental protocols were approved by the Animal Ethics Committee of China Pharmaceutical University (Approval No.: 2024-07-012; Approval date: 27 June 2024).

### 2.3. Establishment of DSS-Induced Experimental Colitis and Folate Administration

Mice were subjected to acute colitis induction via 3% (*w*/*v*) dextran sulfate sodium (DSS; MW 36–50 KDa, MP Biomedicals, Irvine, CA, USA) in autoclaved drinking water ad libitum for 7 days. Disease activity index was monitored by weight loss, stool consistency, and fecal bleeding scoring according to the described protocol [32].

Folate (3 mg/mL, MCE, Trenton, NJ, USA) was dissolved in 1% sodium bicarbonate (Aladdin, Shanghai, China) solution (vehicle) and administered via oral gavage (0.1 mL/10 g) daily during the 7-day DSS exposure period (*n* = 8). Normal control (*n* = 8) and vehicle group (*n* = 8) received equivalent volumes of the vehicle solution. Solutions were freshly prepared before each administration.

### 2.4. Cell Culture and Stimulation Conditions

HT29 and Caco-2 cells were obtained from Procell (Wuhan, China), cultured in McCoy’5A supplemented with 10% FBS (Gibco, Waltham, MA, USA) and DMEM supplemented with 20% FBS, respectively. Cells were stimulated with combined recombinant human TNF-α (30 ng/mL, PeproTech, Cranbury, NJ, USA) and IFN-γ (30 ng/mL, PeproTech, Cranbury, NJ, USA) [33] concurrently treated with 1 µM folate.

### 2.5. Establishment of Caco-2 Monolayer Model and Barrier Integrity Evaluation

Caco-2 cells were seeded onto 0.4-μm pore Transwell inserts (Corning Inc., Corning, NY, USA) at 3 × 10^4^ cells/well and cultured for 21 days to establish polarized monolayers, with medium renewal every 48 h. After modeling and administration, cells were washed with PBS, and transepithelial electrical resistance (TEER) was measured using Millicell^®^ ERS 3.0 Digital Voltohmmeter (Millipore, Burlington, MA, USA). Paracellular permeability was quantified by adding 200 μL of FITC-dextran (1 mg/mL in HBSS, Sigma-Aldrich, St. Louis, MO, USA) to the apical chamber and 600 μL of HBSS to the basolateral chamber. Following 2-h incubation at 37 °C, the basolateral solution was collected, and supernatant fluorescence was measured at 480/520 nm using a microplate reader.

### 2.6. ELISA

Serum Folate levels were determined using the FA/VB9 ELISA Kit (Elabscience, Wuhan, China) according to the manufacturer’s instructions. Similarly, IL-6 and IL-10 in serum were determined using the corresponding ELISA Kit (Biotend, Shanghai, China) according to the manufacturer’s instructions.

### 2.7. Colonic MPO Activity Determination

Colonic MPO activity was determined utilizing the Myeloperoxidase Activity Assay Kit (Elabscience), in accordance with the manufacturer’s protocol.

### 2.8. Histopathological Staining

Distal colonic segments were fixed overnight in 4% paraformaldehyde, dehydrated, paraffin-embedded, sectioned, and stained with hematoxylin & eosin (H&E) and Alcian Blue-Periodic Acid Schiff (AB-PAS) according to the manufacturer’s instructions (Servicebio, Wuhan, China). Representative images were captured using the Nanozoomer S60 (Hamamatsu Photonics, Hamamatsu, Japan). Histological scores were assessed according to previously described standards [32].

### 2.9. Transmission Electron Microscopy (TEM)

Colonic tissues were fixed in 2.5% glutaraldehyde followed by 1% osmium tetroxide, dehydrated through an ethanol series, embedded in resin, sectioned at 70 nm, stained with uranyl acetate/lead citrate, and imaged by transmission electron microscopy (Hitachi HT7800, Hitachi, Tokyo, Japan) to assess the ultrastructural integrity.

### 2.10. Bulk RNA-Sequencing and qRT-PCR

Total RNA of the colon was extracted using the TRIzol reagent (Vazyme, Nanjing, China). For RNA-sequencing, after quantification and quality assessment, libraries were prepared using Illumina TruSeq Stranded mRNA Kit and 150 bp paired-end sequencing performed on NovaSeq 6000 by Gene Denovo (Guangzhou, China). Subsequent differential expression and enrichment analysis were performed on the online platform Omicsmart. For qRT-PCR, after quantification, the obtained RNA was reverse transcribed into cDNA using Hiscript Reverse Transcriptase Kit (Vazyme). qRT-PCR was then performed using One Step qRT-PCR Probe Master Mix (Vazyme) on CFX96 Real-Time PCR cycler (Bio-Rad, Hercules, CA, USA). Relative mRNA expression was determined by the ^ΔΔ^Ct method and normalized to GAPDH. The primers are as follows: GAPDH: (Forward: 5′-AGGTCGGTGTGAACGGATTTG-3′; Reverse: 5′-TGTAGACCATGTAGTTGAGGTCA-3′); IL-1β: (Forward: 5′-GCAACTGTTCCTGAACTCAACT-3′; Reverse: 5′-ATCTTTTGGGGTCCGTCAACT-3′); IL-6: (Forward: 5′-ACAAGTCGGAGGCTTAATTACACAT-3′; Reverse: 5′-TTGCCATTGCACAACTCTTTTC-3′); TNF-α: (Forward: 5′-GACCCTCACACTCAGATCATCTT-3′; Reverse: 5′-CCTTGAAGAGAACCTGGGAGTAG-3′).

### 2.11. Western-Blot

Total proteins extracted by RIPA lysis buffer (Beyotime, Shanghai, China) were quantified, denatured in Laemmli buffer (Bio-Rad), and separated via SDS-PAGE. Proteins were transferred to PVDF membranes, blocked with 5% non-fat milk, and incubated overnight at 4 °C with primary antibodies. After TBST washing, membranes were incubated with HRP-conjugated secondary antibodies (Bioworld, Nanjing, China), developed using ECL substrate (Bio-Rad), and imaged on a ChemiDoc MP system (Bio-Rad). Primary antibodies used are as follows: GAPDH (1:20,000, Abcam, Cambridge, UK), Occludin (1:2000, Proteintech, Wuhan, China), ZO-1 (1:5000, Proteintech), Claudin-1 (1:1000, Proteintech), Claudin-4 (1:1000, Abcam), E-cadherin (1:2000, CellSignalingTechnology, Danvers, MA, USA), β-catenin (1:1000, CellSignalingTechnology), PI3K (1:5000, Zenbio, Chengdu, China), phos-PI3K (1:1000, CellSignalingTechnology), ERK (1:2000, CellSignalingTechnology), phos-ERK (1:2000, CellSignalingTechnology), AKT (1:2000, Proteintech), NF-κB p65 (1:1000, Proteintech), phos-NF-κB p65 (1:1000, CellSignalingTechnology), MLCK (1:1000, Proteintech), MLC2 (1:1000, Proteintech), phos-MLC2 (1:1000, CellSignalingTechnology).

### 2.12. Immunohistochemical and Immunofluorescence Staining

For immunohistochemical, paraffin sections underwent deparaffinization, antigen retrieval, blocking with 5% BSA, and overnight incubation at 4 °C with primary antibodies. After HRP-polymer secondary antibody (1:500, Bioworld, Nanjing, China) incubation, DAB chromogenic development and hematoxylin counterstaining were performed, images were captured by whole-slide scanning using Nanozoomer S60. For immunofluorescence, cells were fixed with ice methanol, followed by blocking with 1% BSA and incubating with primary antibodies overnight at 4 °C. After Alexa Fluor 594-conjugated secondary antibody (1:500, Invitrogen, Carlsbad, CA, USA) and Hoechst (Beyotime) staining, images were obtained using confocal microscope FV3000 (Olympus, Tokyo, Japan). Primary antibodies used are as follows:

ZO-1 (1:500, Proteintech), Occludin (1:200, Proteintech), E-cadherin (1:200, CellSignalingTechnology), Claudin-1 (1:200, Proteintech).

### 2.13. Statistical Analyses

Statistical analyses were performed using GraphPad Prism 9.0. Normality was assessed by the Shapiro–Wilk test. Between-group differences were analyzed using unpaired Student’s *t*-test (two groups) or one-way/two-way ANOVA with Dunnett’s post/Bonferroni’s post hoc test (multiple groups). Specific test methods are reported in the figure legends.

## 3. Results

### 3.1. Inverse Causality Between Circulating Folate and Ulcerative Colitis Risk: A Mendelian Randomization Study

**Figure 1:**
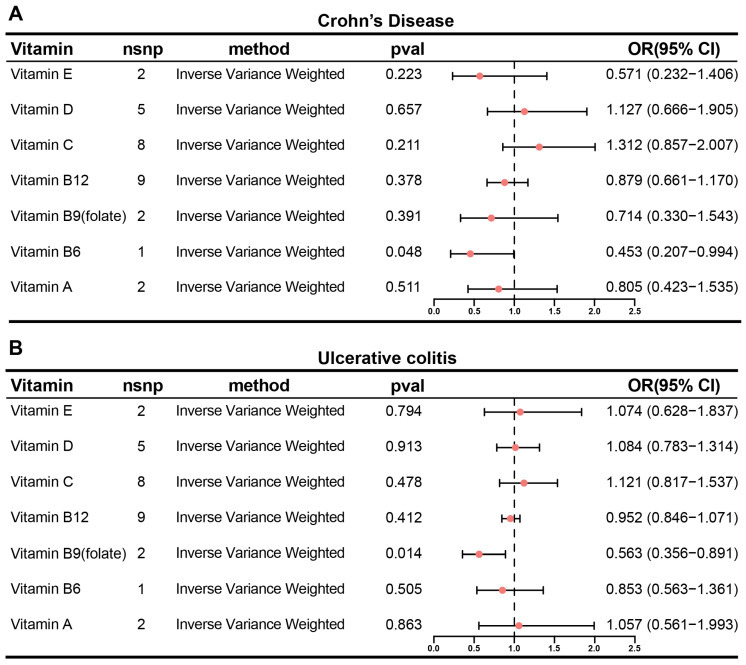
Mendelian randomization analysis of genetically predicted circulating vitamin levels and IBD risk. Forest plots showing the associations between vitamin levels and CD risk (**A**) and UC risk (**B**). *p* values are provided in the figures.

To evaluate the potential causal effects of circulating vitamins on IBD, a two-sample Mendelian randomization analysis was conducted using genetic instruments for vitamins E, D, C, A, B12, B9, and B6, with CD and UC specified as primary outcomes. Inverse variance-weighted (IVW) analysis revealed no significant causal associations between most vitamins and CD risk except for genetically proxied vitamin B6, which demonstrated a nominally significant inverse causal association with reduced disease risk (OR = 0.453, 95% CI = 0.207–0.994; *p* = 0.048) (Figure 1A). Similarly, for UC, genetically proxied vitamin B9 (folate) demonstrated a potential protective role in UC pathogenesis (OR = 0.563, 95% CI = 0.356–0.891; *p* = 0.014) (Figure 1B). Although both vitamins B6 and B9 currently suffer from limited numbers of associated SNPs, the borderline significance and wide confidence interval particularly compromise the statistical robustness of the vitamin B6 association. Therefore, subsequent research will focus on validating the protective effect of folate against UC.

### 3.2. Folate Supplementation Alleviates DSS-Induced Colitis

**Figure 2:**
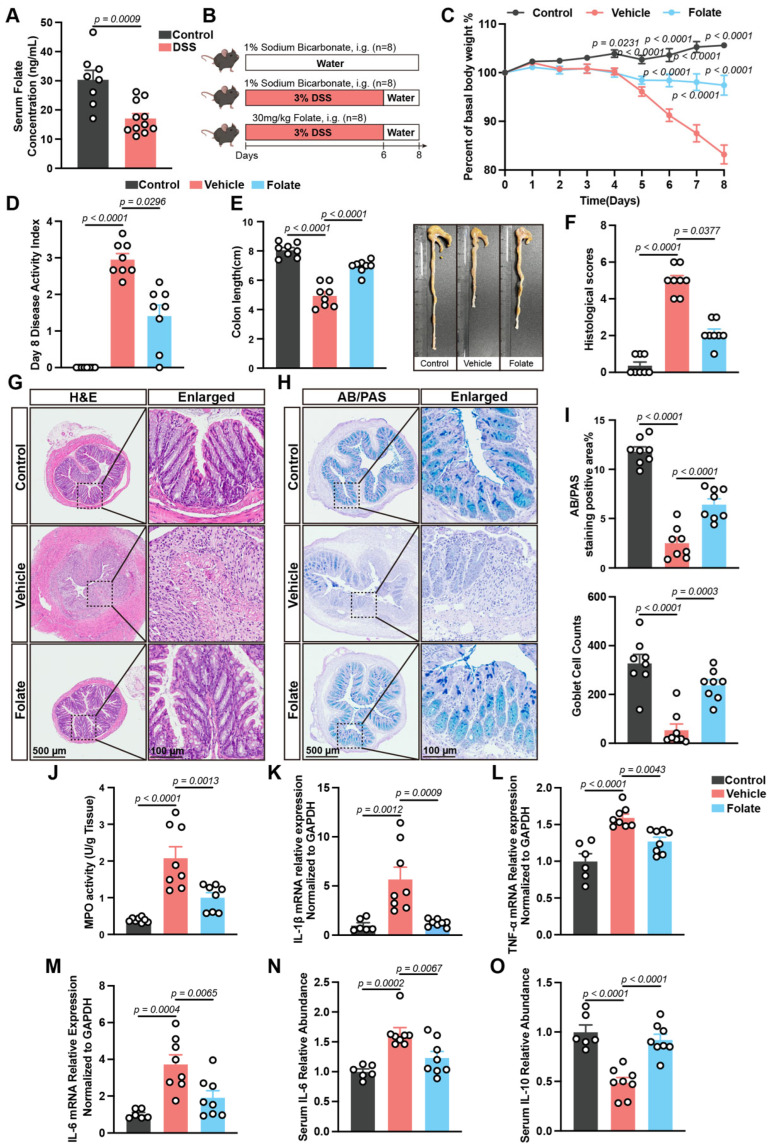
Therapeutic effects of folate supplementation on DSS-induced colitis. (**A**) Serum folate assay (*n* = 8–11). (**B**) Experimental diagram. (**C**) Monitoring of daily weight change (*n* = 8). (**D**) Disease activity index score (*n* = 8). (**E**) Colon length and representative colon images (*n* = 8). (**F**–**I**) Histological analysis of colonic sections. Histological score (*n* = 8) (**F**), H&E staining (**G**), AB/PAS staining (**H**), AB/PAS staining area and goblet cell counts (*n* = 8) (**I**). (**J**) Colonic MPO activity (*n* = 8). (**K**–**M**) Colonic mRNA expression levels of IL-1β (**K**), TNF-α (**L**), and IL-6 (**M**) (*n* = 6–8). (**N**,**O**) Relative abundance of serum IL-6 (**N**) and IL-10 (**O**) (*n* = 6–8). The data are expressed as the mean ± SEM. *p* values are provided in the figures and were determined by unpaired Student’s t test for (**A**); two-way ANOVA for (**C**); one-way ANOVA for (**E**,**I**–**O**); and the Kruskal-Wallis test for (**D**,**F**).

To achieve this verification objective, a DSS-induced experimental colitis model was established, and serum folate assay was initially performed. Notably, serum folate was dramatically decreased in murine colitis, suggesting its potential involvement in UC pathogenesis and indicating that exogenous folate supplementation may offer therapeutic benefits (Figure 2A). Hence, mice with DSS-induced acute colitis were orally administered 30 mg/kg folate (Figure 2B). As expected, compared with the vehicle control, folate intervention significantly improved colitis-associated phenotypes, as evidenced by reduced weight loss, decreased DAI scores, and decreased colon shortening (Figure 2C–E). Additionally, histopathological staining of colonic tissues revealed that DSS stimulation resulted in substantial impairment of the mucosal epithelium and crypt structure integrity, accompanied by extensive infiltration of proinflammatory cells in the submucosal layer. However, folate treatment reversed these pathological symptoms (Figure 2F,G). Moreover, AB-PAS staining was used to assess the structure and function of the intestinal mucus barrier, and the results revealed that the loss of acidic mucus and goblet cells was alleviated after folate administration (Figure 2H,I).

As a well-recognized autoimmune disorder, overactivated mucosal immunity is the core hallmark of UC; thus, the effects of folate on the inflammatory response were investigated. The results revealed that folate markedly decreased MPO activity, a marker of neutrophil infiltration (Figure 2J), and the colonic mRNA expression levels of proinflammatory cytokines, including IL-1β, TNF-α, and IL-6 (Figure 2K–M). Consistently, a reduction in serum IL-6 concentration was observed, while an increase in anti-inflammatory IL-10 concentration was noted following administration (Figure 2N,O). Overall, these results suggest a favorable anti-UC effect of folate.

### 3.3. Folate Restores the Intestinal Epithelial Barrier Both In Vivo and In Vitro

**Figure 3:**
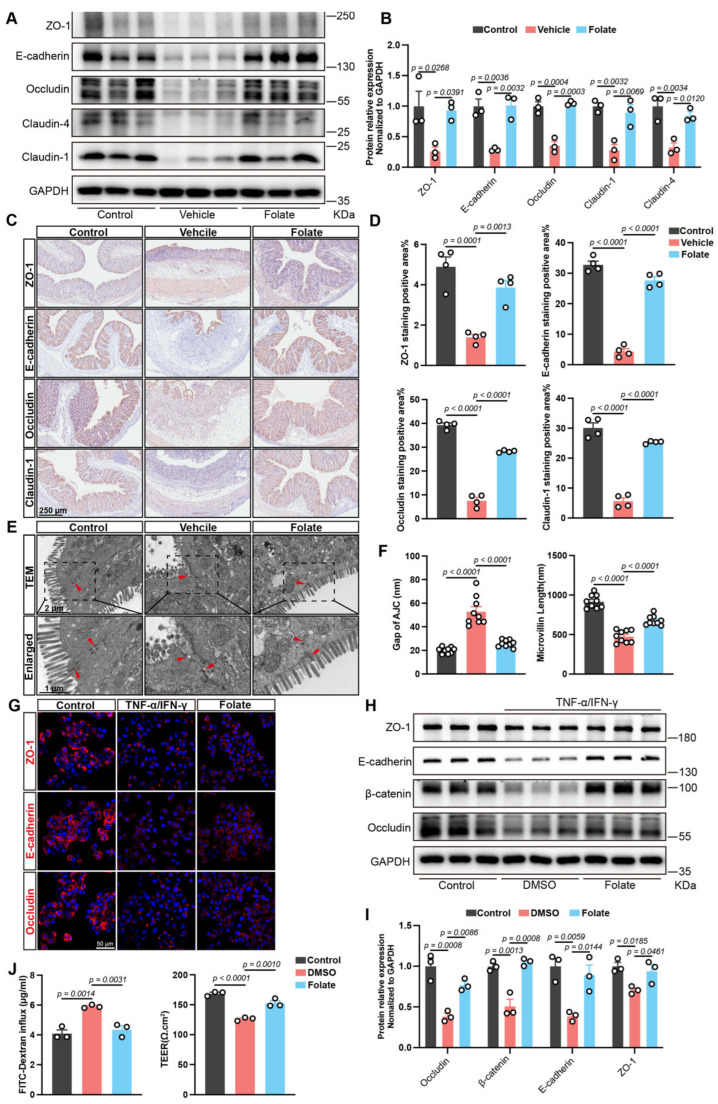
Folate supplementation restored epithelial barrier integrity. (**A**,**B**) Western blot analysis of colonic junction proteins (*n* = 3). (**C**,**D**) Representative images and quantification of immunohistochemical staining for ZO-1, E-cadherin, occludin, and claudin-1 in colonic sections (*n* = 4). (**E**,**F**) Representative TEM images and measurements of the gap in the AJC and length of the microvilli (*n* = 9). (**G**) Representative images of ZO-1, E-cadherin, and occludin expression in TNF-α/IFN-γ-stimulated HT29 cells concurrently treated with 1 µM folate. (**H**,**I**) Western blot analysis of cellular junction proteins (*n* = 3). (**J**) Paracellular permeability and TEER of TNF-α/IFN-γ-stimulated Caco-2 monolayers concurrently treated with 1 µM folate (*n* = 3). The data are expressed as the mean ± SEM. *p* values are provided in the figures and were determined by one-way ANOVA.

In addition to immune dysfunction, impairment of the intestinal epithelial barrier constitutes a critical pathogenic driver in UC. Consequently, the integrity of the epithelial barrier was evaluated by detecting the expression of multiple cellular junctional proteins, including ZO-1, E-cadherin, occludin, and claudins. Immunoblotting revealed that the expression of these proteins was dramatically reduced in the vehicle group compared to control group, whereas folate treatment markedly reversed these changes (Figure 3A,B). Similarly, immunohistochemical staining of colonic tissues showed that folate treatment maintained an intact distribution of these junctional proteins along the mucosal epithelium and crypts (Figure 3C,D). Moreover, TEM analysis of the mucosal epithelium revealed that folate effectively reversed pathological widening of apical junction complex (AJC) gaps and ameliorated the shortening of microvilli, further supporting its role in epithelial barrier repair (Figure 3E,F).

To verify these findings, an in vitro intestinal epithelial barrier damage model was established by treating HT29 cells with recombinant TNF-α and IFN-γ. Immunofluorescence staining demonstrated that 1 µM folate was sufficient to restore the distribution and expression of multiple barrier-associated proteins (Figure 3G), which was verified by quantitative immunoblot analysis (Figure 3H,I). Additionally, a Caco-2 monolayer model confirmed the safeguarding effects of folate on barrier integrity, as it attenuated transepithelial electrical resistance (TEER) reduction and suppressed the increase in dextran permeability (Figure 3J). Collectively, these data indicate the crucial role of folate in ameliorating barrier damage caused by UC.

### 3.4. Folate Reshapes Colonic Transcriptomic Profiles in Colitis Mice

**Figure 4:**
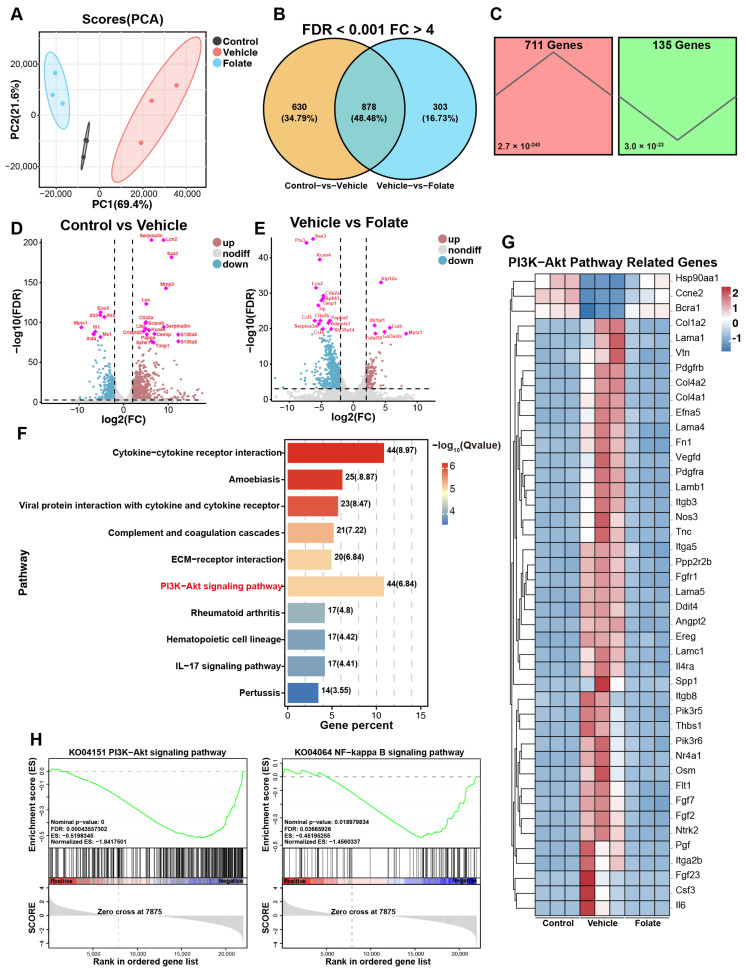
Colonic transcriptomic analysis after folate administration. (**A**) PCA plot. (**B**) Venn diagram. (**C**) Trend analysis of the DEGs. (**D**,**E**) Volcano plots. (**F**) KEGG analysis of DEGs. (**G**) Heatmap of DEGs associated with the PI3K/AKT pathway. (**H**) GSEA plots of the PI3K/AKT and NF-κB pathways.

To elucidate the molecular mechanisms underlying folate-mediated epithelial barrier restoration, an RNA-sequencing analysis of colonic tissue was conducted. The PCA shift along PC1 demonstrated the capacity of folate to globally reverse pathogenic transcriptomic networks in colitis (Figure 4A). Differentially expressed genes (DEGs) were identified using stringent thresholds (|fold change| > 4; adjusted *p* value (FDR) < 0.001) and further refined by Venn diagram intersection analysis (Figure 4B; for a complete list, see Supplementary Materials File S2). Among these core genes, 711 were upregulated in the colitis model and subsequently downregulated by folate treatment, while 135 genes exhibited reciprocal expression patterns (Figure 4C–E). Given the magnitude of the DEGs, KEGG pathway enrichment analysis was performed. In addition to the expected cytokine signaling pathways, significant enrichment of the PI3K/AKT pathway was observed (Figure 4F). These findings prompted a systematic reexamination of the expression changes in all 44 PI3K/AKT-associated DEGs, which revealed a near-universal downregulation after folate administration (Figure 4G). Gene set enrichment analysis (GSEA) further confirmed the suppression of both the PI3K/AKT pathway and its downstream NF-kappa B signaling cascade (Figure 4H), demonstrating folate-mediated inhibition of this proinflammatory axis. Collectively, the results of the transcriptomic profiling suggest that the PI3K/AKT/NF-κB axis is suppressed in folate-mediated barrier restoration, suggesting that its potential as a mechanistic pathway warrants further investigation.

### 3.5. Involvement of PI3K/AKT/NF-κB Pathway Inhibition in Folate-Mediated Barrier Restoration

**Figure 5:**
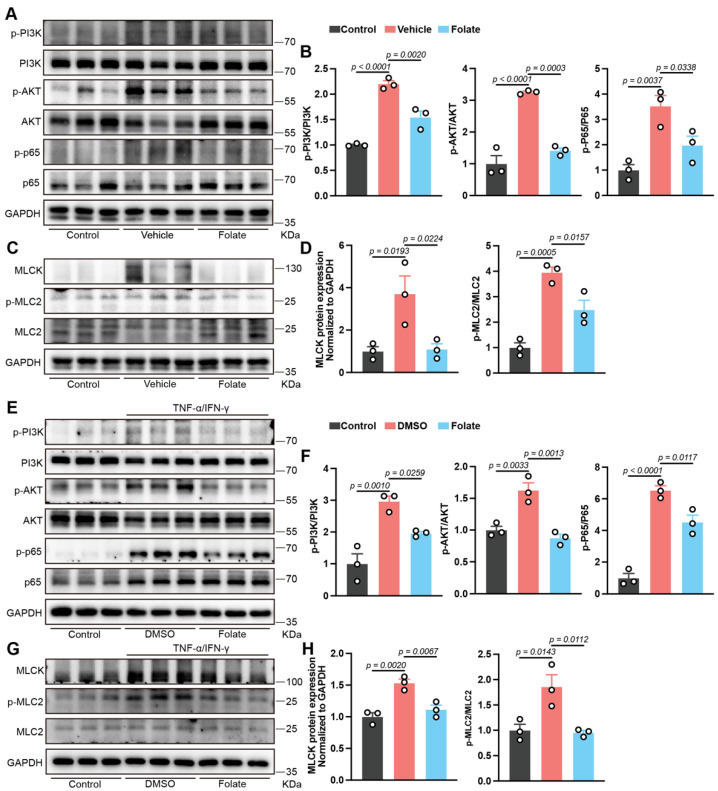
Folate inhibits the PI3K/AKT/NF-κB/MLCK/MLC2 axis. (**A**,**B**) Immunoblotting analysis of colonic PI3K, AKT, and NF-κB p65 levels and their phosphorylation states (*n* = 3). (**C**,**D**) Immunoblotting analysis of colonic MLCK, MLC2 and phos-MLC2 (*n* = 3). (**E**,**F**) Immunoblotting analysis of cellular levels of PI3K, AKT, and NF-κB p65 and their phosphorylation states (*n* = 3). (**G**,**H**) Immunoblotting analysis of cellular MLCK, MLC2 and phos-MLC2 (*n* = 3). *p* values are provided in the figures and were determined by one-way ANOVA.

Consequently, the protein content and phosphorylation status of the PI3K, AKT, and NF-κB p65 subunits were subsequently assessed. Consistent with the results of the transcriptomic predictions, immunoblotting results confirmed the folate-mediated suppression of this signaling cascade, as evidenced by significantly reduced phosphorylation ratios (p-protein/total) across PI3K, AKT, and NF-κB p65 following treatment (Figure 5A,B). As a transcriptional target of NF-κB p65, myosin light chain kinase (MLCK) phosphorylates myosin light chain 2 (MLC2), culminating in junctional protein degradation and structural compromise of the AJC [34]. Notably, attenuated activation of the MLCK/MLC2 pathway was also observed (Figure 5C,D). Furthermore, folate suppressed the PI3K/AKT/NF-κB/MLCK/MLC2 signaling cascade in vitro, thereby corroborating the in vivo findings (Figure 5E–H). These results establish that the inhibition of this pathway is a key mechanism through which folate contributes to epithelial barrier restoration.

## 4. Discussion

Folate, a core component of one-carbon metabolism essential for DNA synthesis, is well-established for its role in preventing anemia, hypertension, neuropsychiatric disorders, and reducing cancer risk [35]; however, its relationship with IBD risk remains incompletely defined. In the present study, Mendelian randomization (MR) analysis revealed an inverse causal effect of elevated circulating folate levels on UC risk, indicating a protective, disease-modifying role of folate. These findings align with and strengthen existing MR evidence and meta-analytic data supporting the protective effect of folate on UC [25,36,37]. Clinically, folate deficiency is highly prevalent in IBD patients, particularly among those receiving sulfasalazine or methotrexate therapy [38]. Consistently, hyperhomocysteinemia in IBD patients potentiates intestinal inflammation, whereas experimental folate deprivation in low-methyl-donor diets exacerbates colitis severity [39,40]. Based on these findings, we proposed that exogenous folate supplementation could have therapeutic effects on UC, which was subsequently validated in an established DSS-induced colitis model.

The intestinal barrier, comprising the epithelial barrier and mucus layer, serves as the primary defense interface, separating the internal milieu from the luminal environment. This dynamic structure facilitates crucial interactions between the mucosal immune system and luminal antigens, playing a pivotal role in maintaining intestinal homeostasis [41]. Although immune dysregulation is widely regarded as a central mechanism in IBD, current immunosuppressive therapies demonstrate limited efficacy and carry significant risks of infection and malignancy [42], suggesting that the primacy of immune factors in IBD pathogenesis may be overstated. Notably, emerging clinical evidence highlights intestinal barrier disruption as a pivotal factor in IBD development. Barrier defects have been shown to precede clinical symptom onset, while the restoration of barrier integrity correlates with deeper mucosal healing and improved long-term prognosis [43,44]. Consequently, pursuing barrier repair strategies beyond immunosuppression represents an emerging frontier in IBD therapeutic development. Our findings demonstrate that folate supplementation robustly restores the structural and functional integrity of the intestinal epithelial barrier both in vivo and in vitro injury models, suggesting folate as a promising barrier-targeted therapeutic agent for mucosal healing. RNA sequencing was utilized to investigate the molecular mechanism underlying folate-mediated barrier restoration, with integrated pathway enrichment analysis of DEGs revealing significant suppression of the PI3K/AKT/NF-κB signaling pathway following folate intervention. Cytoskeletal rearrangement, a key mechanism in intestinal epithelial barrier pathogenesis, involves MLCK—an IBD-associated molecule and promising drug target. As a canonical NF-κB p65 target gene, MLCK is significantly upregulated under pathological conditions and translocates to the perijunctional actomyosin ring, where it disrupts AJC integrity and increases paracellular permeability [45]. However, the ubiquitous expression of MLCK in multiple organ systems (e.g., cardiac and skeletal muscle) results in unavoidable cardiotoxicity and myotoxicity of its inhibitors, thereby impeding clinical translation [46]. By inhibiting the PI3K/AKT/NF-κB pathway, folate significantly downregulated MLCK expression and subsequent MLC2 phosphorylation, demonstrating that this signaling axis mediates its restorative effect on the epithelial barrier. Given this mechanism, folate presents a favorable safety advantage over direct MLCK inhibitors, highlighting its translational potential for barrier repair therapies in UC. Nevertheless, the deeper mechanistic actions of folate in the complex pathophysiology of UC remain to be fully characterized. Key unresolved issues include determining whether folate modulates PI3K/AKT signaling primarily through its receptor or other target proteins, as well as elucidating the crosstalk between folate-mediated one-carbon metabolism in epithelial cells and the local immune microenvironment.

Notably, our MR analysis did not reveal a significant association between vitamin D levels and IBD risk, which contrasts with the prevailing consensus regarding its protective role in IBD [19]. This discrepancy likely reflects the unresolved question of causality—namely, whether vitamin D deficiency contributes to IBD development or arises as a consequence of the disease [47]. While vitamin D deficiency is both prevalent in IBD patients and is a known risk factor for disease [37,48], several studies have reported no significant association between serum vitamin D levels and IBD development or disease activity [49,50]. Furthermore, despite robust efficacy in animal models [19,51,52], clinical evidence remains limited to modest reductions in hospitalization rates and corticosteroid use demonstrated in recent human studies [53]. Consequently, our findings do not refute the established protective role of vitamin D in IBD, but these controversies underscore the persistent need for large-scale prospective trials to validate the therapeutic efficacy of supplementation. Similarly, conclusions regarding other vitamins are constrained by the inherent limitations of MR, such as the limited number of available genetic variants and potential confounding by population stratification. Therefore, our findings do not definitively preclude a potential biological role for other vitamins such as B1 and B5 in UC, especially given supportive evidence from existing preclinical studies [21,22].

Collectively, the results of this study demonstrate that elevated circulating folate levels protect against UC development and that folate supplementation restores intestinal barrier integrity by suppressing the PI3K/AKT/NF-κB/MLCK/MLC2 axis across experimental models. Furthermore, the well-established safety profile of folate, evidenced by its history in public health fortification without significant toxicity even at high intake levels [54,55], highlights the strong therapeutic potential and translational feasibility of folate supplementation as a novel strategy for UC management. Building upon this favorable safety profile, our preclinical dosing strategy was designed to compensate for the low intestinal distribution of folate, a primary pharmacokinetic constraint [56]. Consequently, the clinical translation of this strategy necessitates future work either to establish a safe and effective systemic dosing regimen in clinical trials, or to develop colon-targeted formulations that enhance local exposure, thereby reducing the required dose and improving the therapeutic safety profile. To this end, subsequent research should pursue large-scale clinical trials to gather real-world evidence for clarifying the causal role of folate deficiency in UC etiology and quantifying the therapeutic benefit of supplementation, thereby bridging preclinical findings to evidence-based clinical applications.

## 5. Conclusions

Guided by Mendelian randomization analysis, this study demonstrates that genetically predicted high circulating folate levels confer protection against UC. Experimentally, exogenous folate supplementation significantly ameliorated DSS-induced colitis and restored inflammation-disrupted intestinal epithelial barrier integrity both in vivo and in vitro. Mechanistically, these effects were mediated through suppression of the PI3K/AKT/NF-κB/MLCK/MLC2 signaling axis. Collectively, our findings provide novel insights into UC pathogenesis and suggest promising translational opportunities for folate-based therapeutic strategies.

## Data Availability

The data that support the findings of this study are available from the corresponding author upon reasonable request.

## Supplementary Materials

The following supporting information can be downloaded at: https://www.mdpi.com/article/10.3390/biology14111573/s1, Supplementary Materials File S1: Details of genetic instruments and sensitivity analyses; Table S1: summary of instrumental variable SNPs; Figures S1–S6: Sensitivity analyses; Supplementary Materials File S2: Summary of DEGs; Supplementary Materials File S3: Origin images for blots.
