# Supplementary material for: Folate Attenuates Ulcerative Colitis via PI3K/AKT/NF-κB/MLCK Axis Inhibition to Restore Intestinal Barrier Integrity
Source: Biology (Basel). 2025 Nov 10;14(11):1573. doi: 10.3390/biology14111573 (PMC12650468; doi:10.3390/biology14111573)
Supplement: Supplementary file 1 [file biology-14-01573-s001.zip › Supplementary materials file S1.pdf]

Table S1 Summary of SNPs used as instrumental variables for circulating vitamin levels

Exposure	SNPs	EA	OEA	SE	Beta	EAF	*p*-value
Vitamin A	rs10882272	C	T	0.015	-0.087	0.38	7.00E-15
Vitamin A	rs1667255	C	A	0.015	0.090	0.37	6.00E-14
Folate	rs1801133	G	A	0.008	0.096	0.67	9.50E-53
Folate	rs652197	C	T	0.010	0.070	0.18	1.40E-12
Vitamin B6	rs4654748	T	C	0.280	1.450	0.52	8.30E-18
Vitamin B12	rs3742801	T	C	0.009	0.045	0.29	1.70E-13
Vitamin B12	rs602662	A	G	0.007	0.160	0.60	2.40E-139
Vitamin B12	rs34324219	C	A	0.007	0.210	0.88	1.10E-11
Vitamin B12	rs2336573	T	C	0.007	0.320	0.03	8.40E-59
Vitamin B12	rs41281112	C	T	0.020	0.170	0.95	8.90E-35
Vitamin B12	rs12272669	A	G	0.007	0.510	0.01	3.00E-09
Vitamin B12	rs56077122	A	C	0.009	0.087	0.34	4.80E-21
Vitamin B12	rs1801222	G	A	0.007	0.110	0.59	3.30E-75
Vitamin B12	rs1131603	C	T	0.017	0.190	0.06	4.90E-49
Vitamin C	rs6693447	T	G	0.006	0.039	0.55	6.25E-10
Vitamin C	rs10051765	C	T	0.007	0.039	0.34	3.64E-09
Vitamin C	rs13028225	T	C	0.009	0.102	0.86	2.38E-30
Vitamin C	rs9895661	T	C	0.008	0.063	0.82	1.05E-14
Vitamin C	rs10136000	A	G	0.007	0.040	0.28	1.33E-08
Vitamin C	rs33972313	C	T	0.018	0.360	0.97	4.61E-90
Vitamin C	rs2559850	A	G	0.006	0.058	0.60	6.30E-20
Vitamin C	rs117885456	A	G	0.012	0.078	0.09	1.70E-11
Vitamin D	rs17216707	C	T	0.008	-0.080	0.21	8.14E-23
Vitamin D	rs3755967	T	C	0.008	-0.270	0.28	1.00E-200
Vitamin D	rs10741657	G	A	0.007	-0.090	0.60	2.05E-46
Vitamin D	rs12785878	G	T	0.007	-0.110	0.25	3.80E-62
Vitamin D	rs10745742	C	T	0.007	-0.050	0.60	1.88E-14
Vitamin E	rs2108622	T	C	0.025	0.072	0.14	1.00E-14
Vitamin E	rs11057830	A	G	0.028	0.082	0.28	8.00E-09

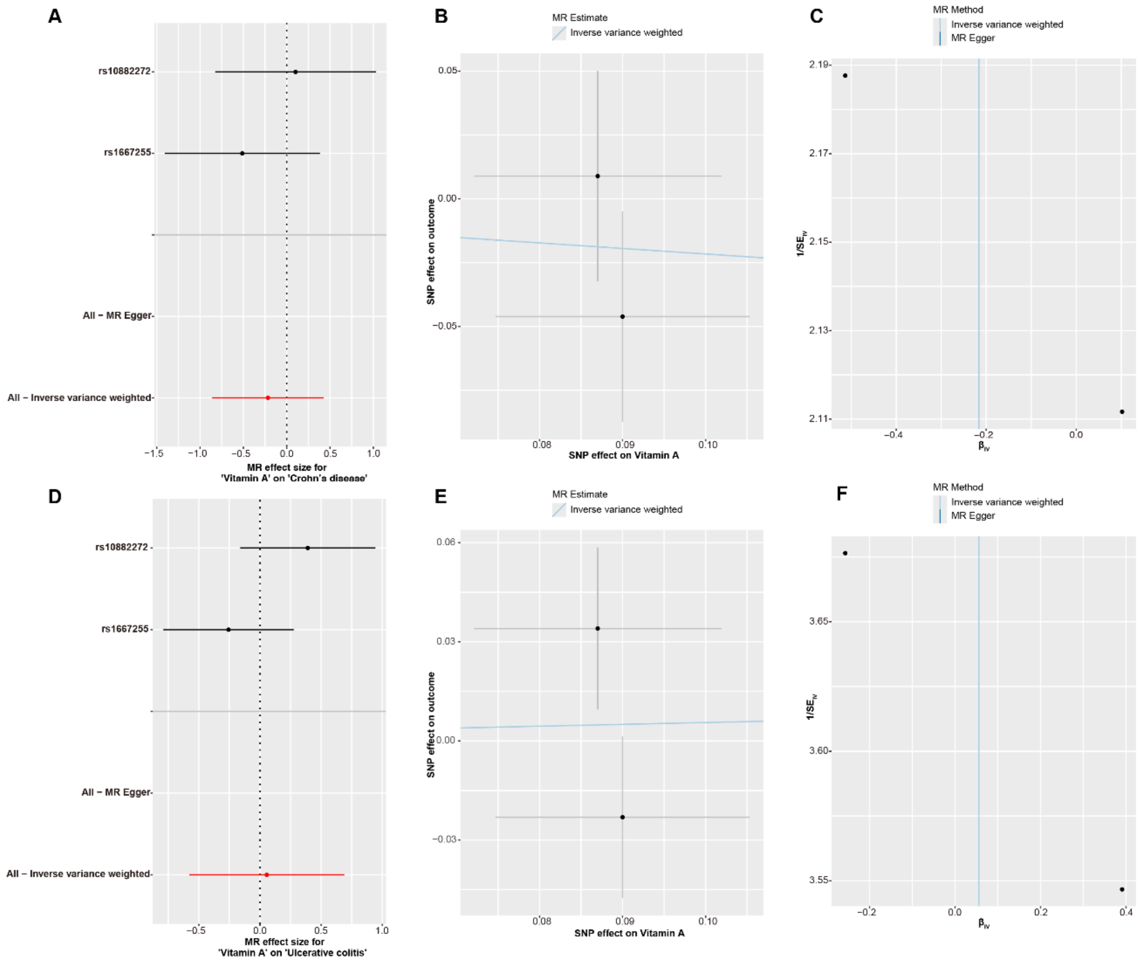

Figure S1 Sensitivity analysis of the causal effect of vitamin A on CD and UC using Mendelian randomization. (**A**) Forest plot, (**B**) scatter plot, and (**C**) funnel plot for CD. (**D**) Forest plot, (**E**) scatter plot, and (**F**) funnel plot for UC. Insufficient number of SNPs for leave-one-out analysis.

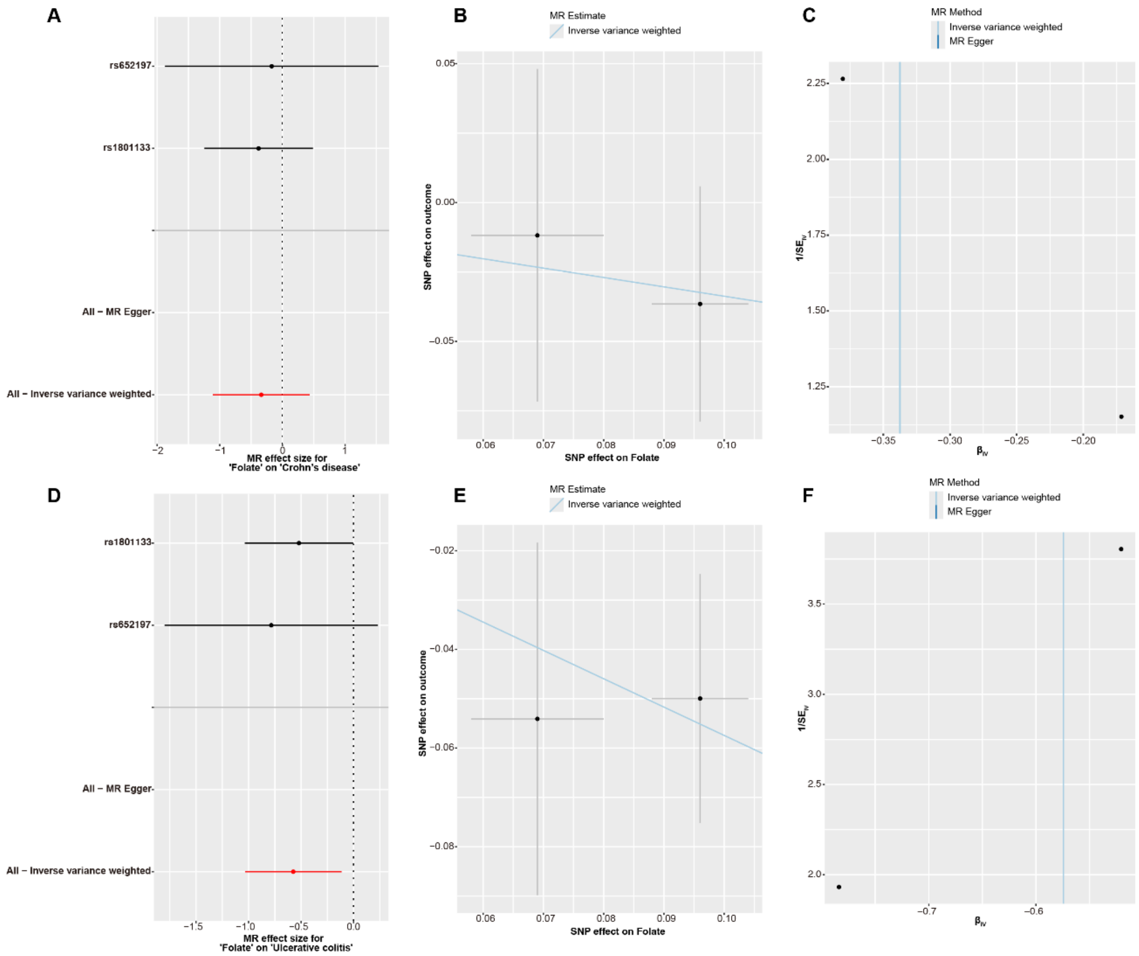

Figure S2 Sensitivity analysis of the causal effect of folate on CD and UC using Mendelian randomization. (**A**) Forest plot, (**B**) scatter plot, and (**C**) funnel plot for CD. (**D**) Forest plot, (**E**) scatter plot, and (**F**) funnel plot for UC. Insufficient number of SNPs for leave-one-out analysis.

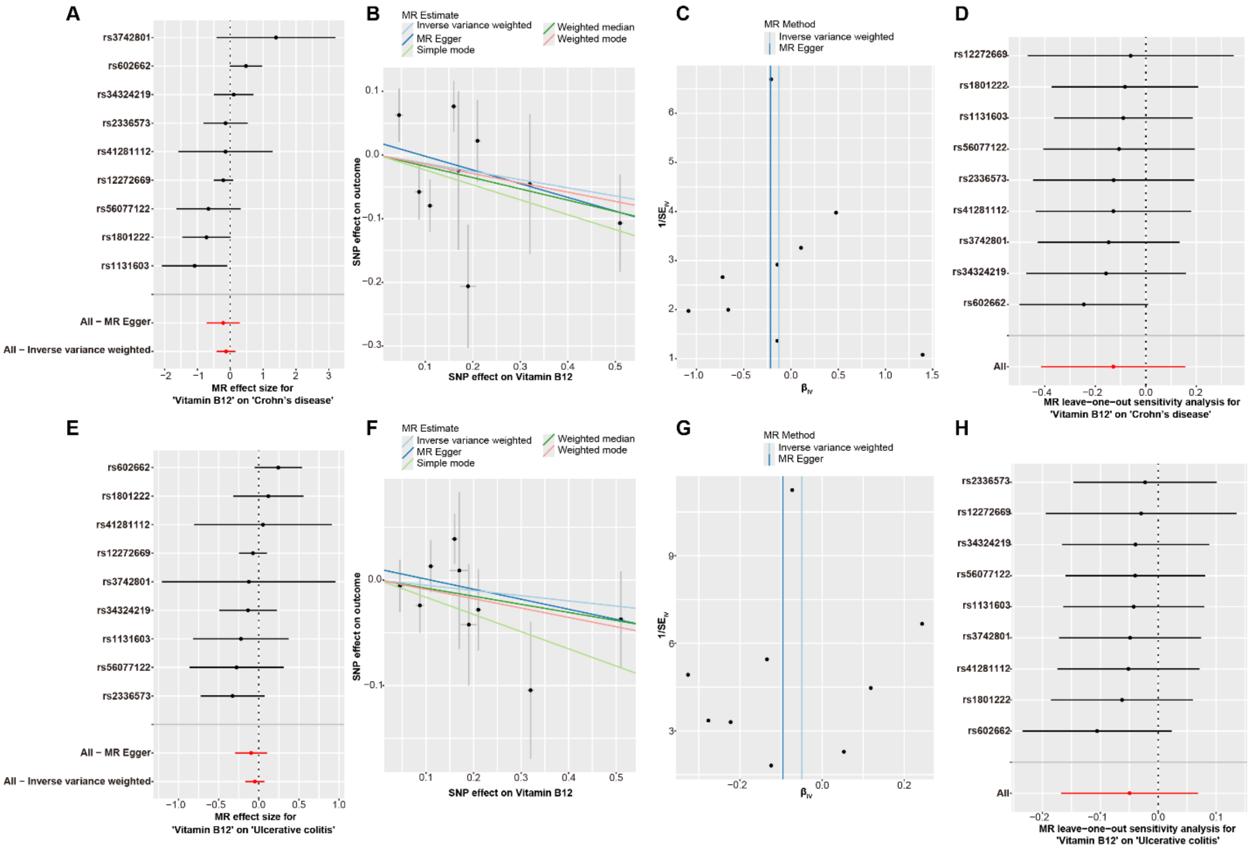

Figure S3 Sensitivity analysis of the causal effect of vitamin B12 on CD and UC using Mendelian randomization. (**A**) Forest plot, (**B**) scatter plot, and (**C**) funnel plot, and (**D**) leave-one-out plot for CD. (**E**) Forest plot, (**F**) scatter plot, (**G**) funnel plot, and (**H**) leave-one-out plot for UC.

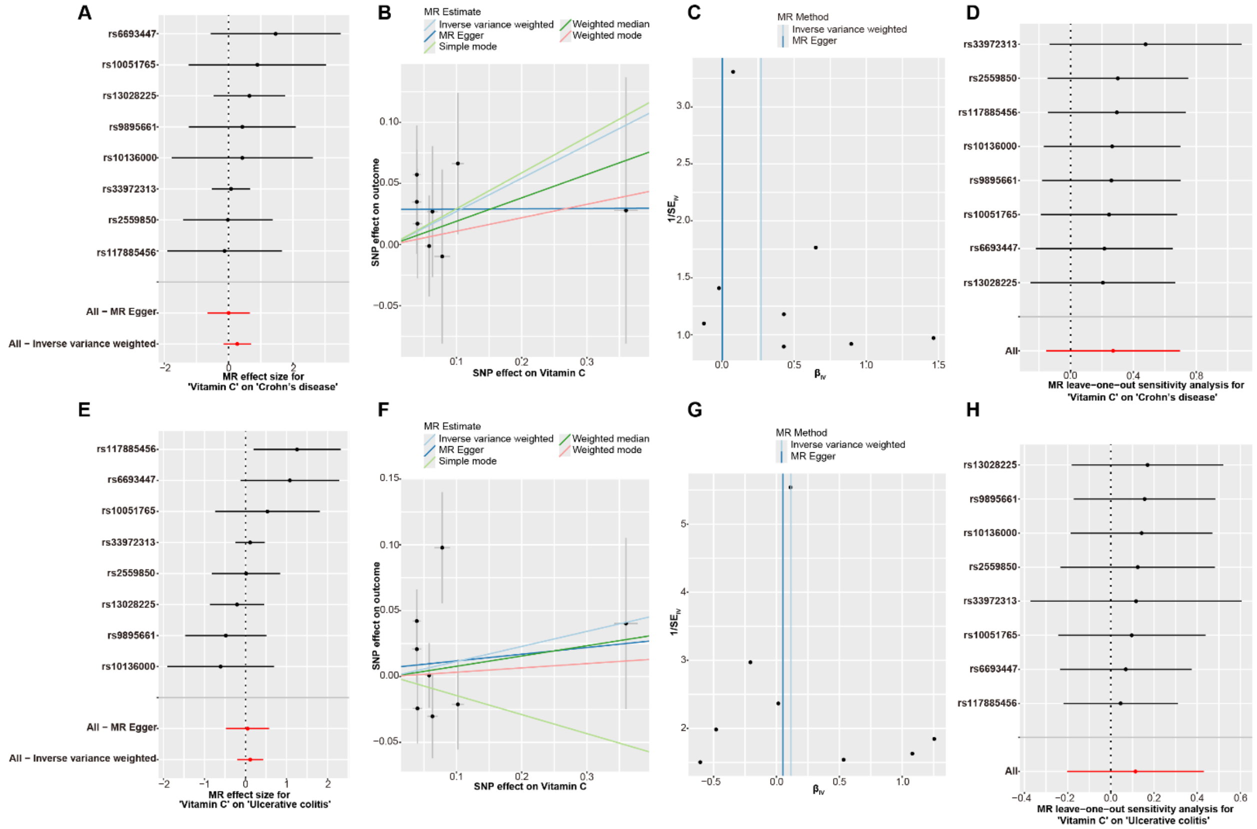

Figure S4 Sensitivity analysis of the causal effect of vitamin C on CD and UC using Mendelian randomization. (**A**) Forest plot, (**B**) scatter plot, and (**C**) funnel plot, and (**D**) leave-one-out plot for CD. (**E**) Forest plot, (**F**) scatter plot, (**G**) funnel plot, and (**H**) leave-one-out plot for UC.

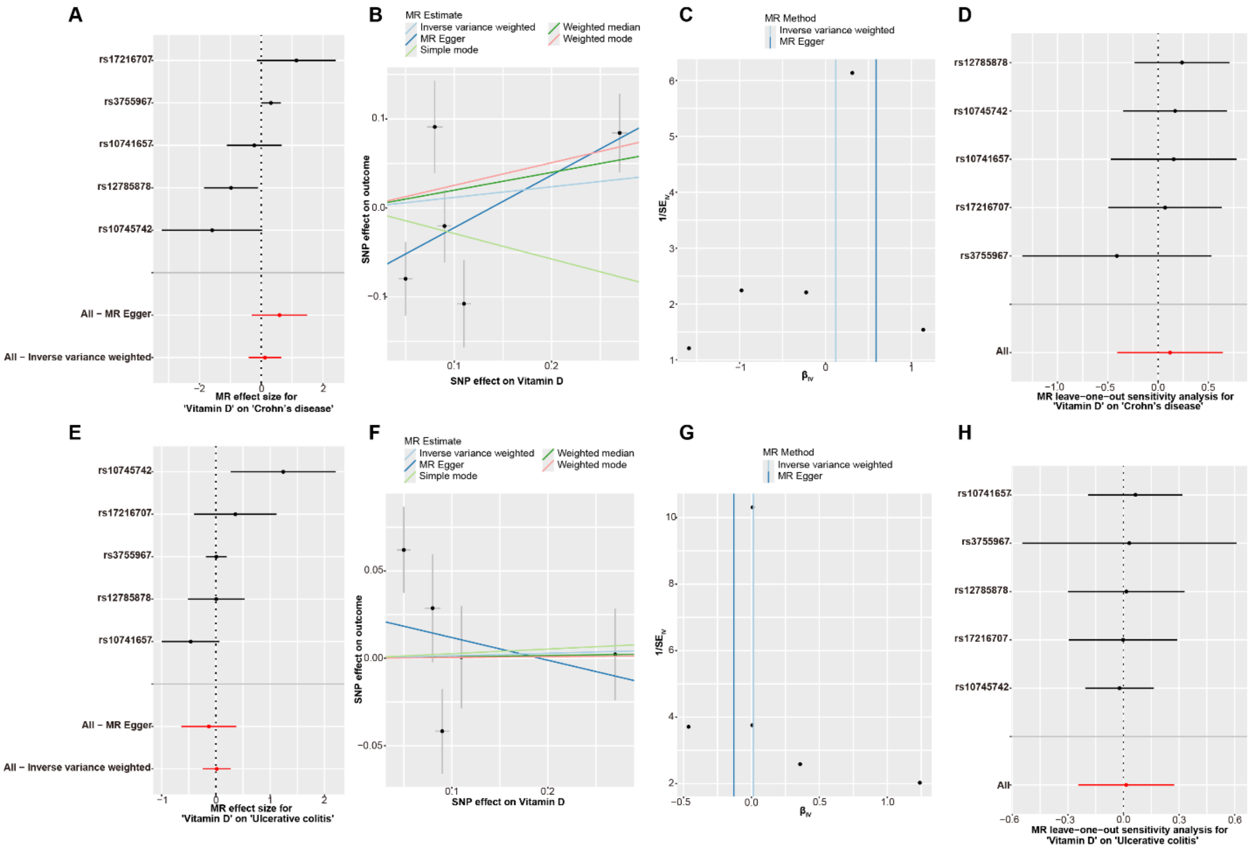

Figure S5 Sensitivity analysis of the causal effect of vitamin D on CD and UC using Mendelian randomization. (**A**) Forest plot, (**B**) scatter plot, and (**C**) funnel plot, and (**D**) leave-one-out plot for CD. (**E**) Forest plot, (**F**) scatter plot, (**G**) funnel plot, and (**H**) leave-one-out plot for UC.

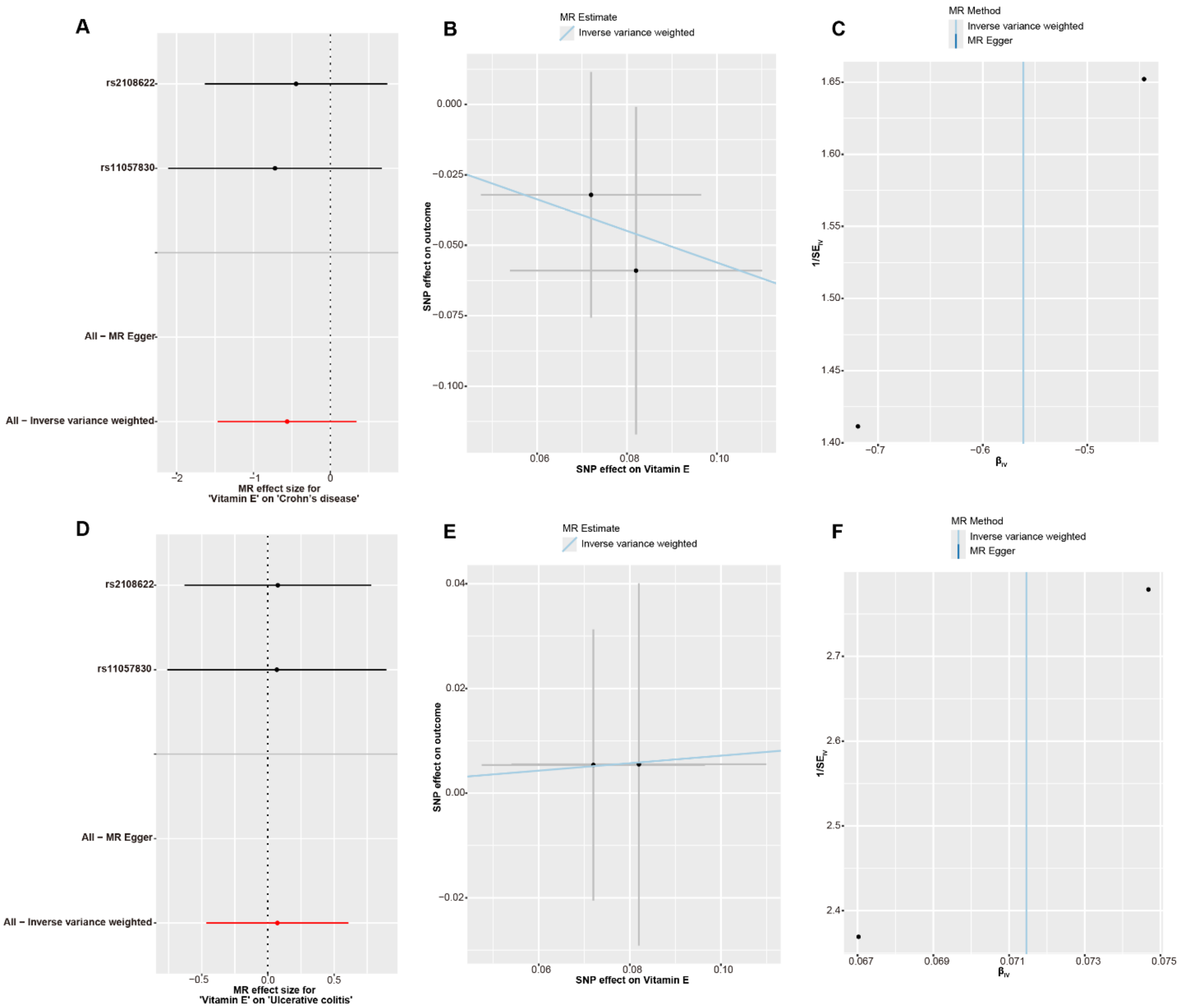

Figure S6 Sensitivity analysis of the causal effect of vitamin E on CD and UC using Mendelian randomization. (**A**) Forest plot, (**B**) scatter plot, and (**C**) funnel plot for CD. (**D**) Forest plot, (**E**) scatter plot, and (**F**) funnel plot for UC. Insufficient number of SNPs for leave-one-out analysis.
