# Supplementary figures and images for: Folate Attenuates Ulcerative Colitis via PI3K/AKT/NF-κB/MLCK Axis Inhibition to Restore Intestinal Barrier Integrity
Source: Biology (Basel). 2025 Nov 10;14(11):1573. doi: 10.3390/biology14111573 (PMC12650468; doi:10.3390/biology14111573)

Figure 3A

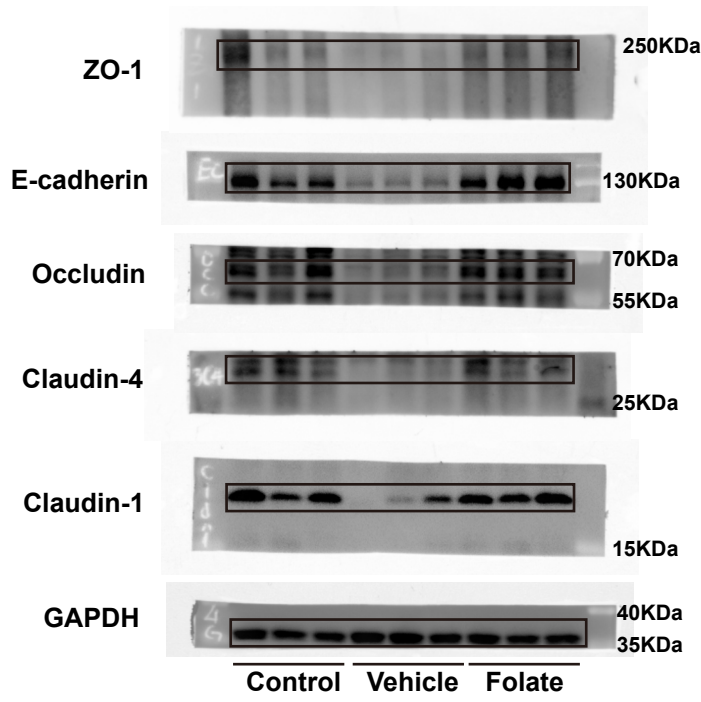

Figure 3H

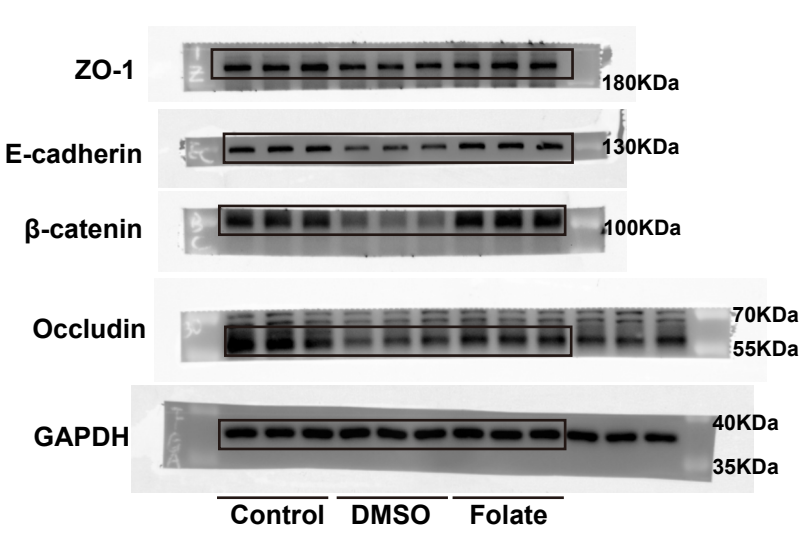

Figure 5A

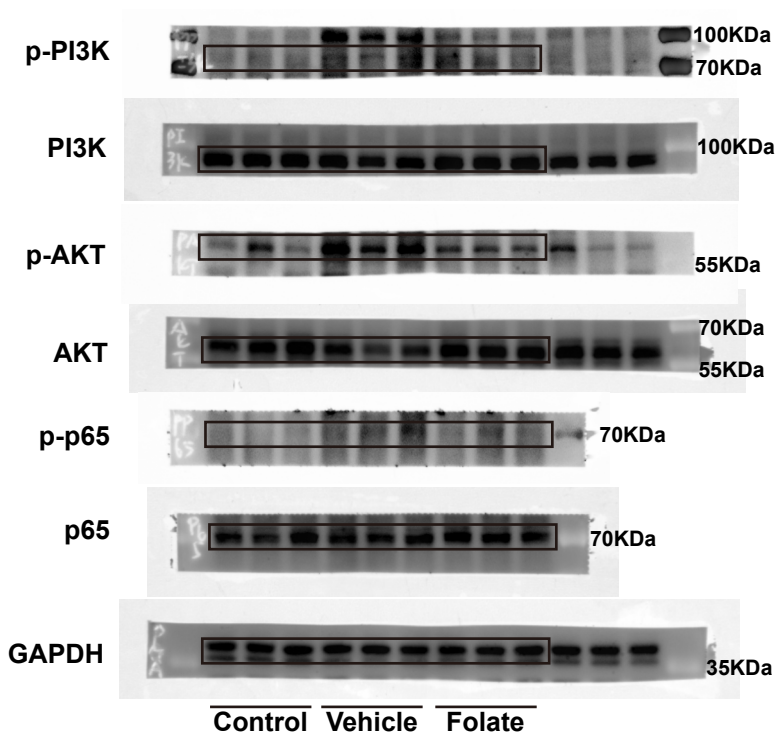

Figure 5E

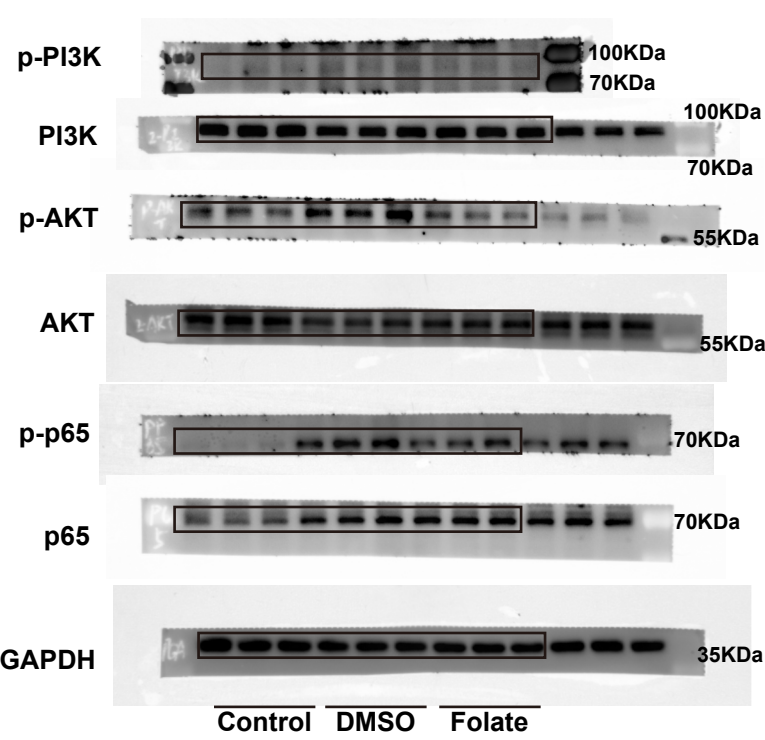

Figure 5C

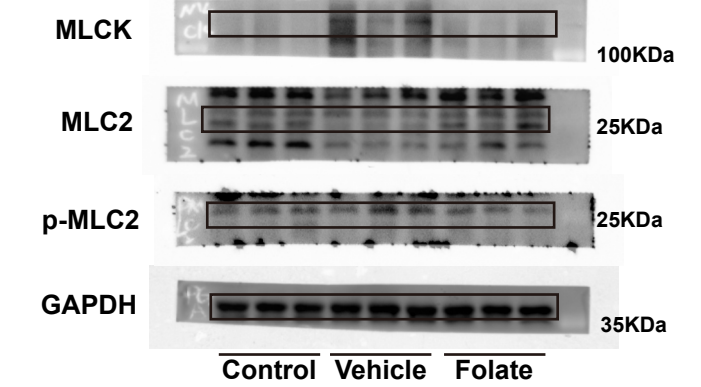

Figure 5G

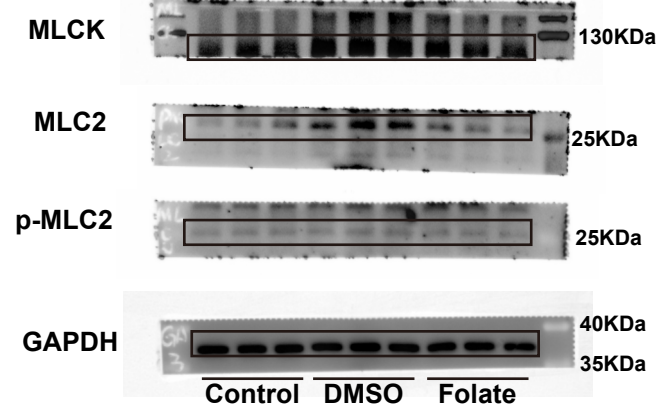

Supplement: Supplementary file 1 [file biology-14-01573-s001.zip › Supplementary materials file S3.pdf]
